# Patient Perspectives on Trust in Artificial Intelligence–Powered Tools in Prostate Cancer Diagnostics

**DOI:** 10.1177/10497323251387545

**Published:** 2025-11-18

**Authors:** Simon Aleksander Berger, Erna Håland, Marit Solbjør

**Affiliations:** 1 Department of Education and Lifelong Learning, Faculty of Social and Educational Sciences, 8018 Norwegian University of Science and Technology, Trondheim, Norway; 2 Department of Public Health and Nursing, Faculty of Medicine and Health Sciences, 8018 Norwegian University of Science and Technology, Trondheim, Norway

**Keywords:** artificial intelligence, trust, prostate cancer diagnostics, users’ experiences, doctor–patient communication, patient safety, qualitative interviews, reflexive thematic analysis, Norway

## Abstract

The increasing prevalence of prostate cancer cases calls for new ways to improve diagnostic pathways and patient care. Artificial intelligence (AI)–powered tools offer potential to streamline prostate cancer diagnostics, yet little is known about how patients perceive and experience these technologies. This study investigates how patients diagnosed through an AI-powered prostate cancer diagnostic tool express trust in AI. Based on 18 semi-structured qualitative interviews with men who underwent AI-supported diagnostics, data were analyzed using reflexive thematic analysis. Three dimensions of trust were developed. First, participants described a foundational trust in the healthcare system and professionals, shaped by previous positive encounters. Second, this interpersonal trust was central to their acceptance of AI, as participants relied on healthcare professionals and their expertise to explain, justify, and endorse AI technology. Third, participants recognized AI’s potential to enhance diagnostics but emphasized the demand for human oversight and second opinion, due to concerns about accountability and AI’s lack of intuition and holistic clinical oversight. Participants were more forgiving of human errors than those made by AI, highlighting the relational and moral dimensions of trust in healthcare. These findings underscore the importance of human relationships in shaping how patients engage with AI technologies and how trust in healthcare professionals remains a critical mediator for acceptance of AI in clinical settings. Understanding patients’ trust in AI requires attention to the sociotechnical context in which care is delivered. In conclusion, trust in healthcare professionals remains paramount and is crucial for the acceptance of AI in prostate cancer diagnostics.

## Introduction

Prostate cancer is the most frequently diagnosed cancer in men worldwide, accounting for 15% of cancer cases (James et al., 2024). The Lancet Commission on prostate cancer suggests a surge in new prostate cancer cases annually, rising from 1–4 million in 2020 to 2–9 million by 2040 (James et al., 2024). This increase puts pressure on the healthcare sector’s capacity to screen, diagnose, and treat patients. Streamlining cancer diagnostics and treatment can alleviate the need for healthcare resources. Novel artificial intelligence (AI)–powered tools offer new ways to screen and diagnose, contributing to the streamlining of cancer care.

A growing field of research focuses on challenges with the introduction of AI tools and how this implementation can change radiologists’ working practices (Thomas et al., 2023; Wenderott et al., 2024). Fewer studies focus on patients’ expectations for AI (Fransen et al., 2025; Lysø et al., 2024). Extensive knowledge about societal implications of large-scale implementation of AI in healthcare is currently lacking (Alami et al., 2024; Alowais et al., 2023; Reddy, 2024), especially research on how AI implementation may impact patient care (Reddy, 2024). Lysø et al. (2024) emphasize the importance of including prostate cancer patients in research on implementation and use of AI, as they are the ultimate end-users. As the number of men who have undergone prostate cancer diagnostics with assistance of AI is limited, most previous research is based on prostate cancer patients and men in general. Thus, previous research on patients with real experiences from AI-assisted prostate cancer diagnostics is limited. Our study contributes to the research field by investigating experiences of men who have been diagnosed with prostate cancer by an AI-powered tool.

### AI in Prostate Cancer Diagnostics

In cancer treatment, magnetic resonance imaging (MRI) is one of the main tools for diagnosis, consequently producing massive amounts of medical images (Bi et al., 2019; van Leeuwen et al., 2022). Interpreting these images is a time-consuming task for healthcare professionals, occupying large resources (van Leeuwen et al., 2022), and time spent interpreting these pictures is likely to increase with rising numbers of prostate cancer cases, calling for new and better solutions. New technologies such as machine learning and AI-powered diagnostic tools are expected to shorten medical image reading time and ease radiologists’ workload caused by the rising number of cancers, including prostate cancer, and are generally expected to improve healthcare (Fransen et al., 2025; Lysø et al., 2024; Nilsen et al., 2022; Solberg & Kirchhoff, 2023; van Leeuwen et al., 2022; Wenderott et al., 2024).

Understanding technology’s ability to affect us even when it is not in use (Kiran, 2012) can help us recognize the effect AI may have on patients and society. Technology’s ability to affect us irrespective of its use should not be considered a technology deterministic perspective but rather a sociotechnical one, as technology is developed through social processes (Latour, 1991). Increased availability of digital devices and constant access to internet changes our ways of communicating simply by existing (Kiran, 2012). AI’s omnipresence in public discourse can also influence patients’ views on AI (Cacciatore et al., 2015).

Previous research on prostate cancer patients’ perception of AI in prostate cancer diagnostics presents a predominantly favorable view (Fransen et al., 2025; Lysø et al., 2024). For instance, patients expect that AI will improve the diagnostic process by providing more precise diagnoses earlier (Lysø et al., 2024). Prostate cancer patients are more willing to accept autonomous AI decisions if AI’s performance is proven to outperform radiologists, but they still prefer radiologists to provide second opinions (Fransen et al., 2025). Several comprehensive international validation studies find that AI already can correctly identify and grade medical images with the same accuracy as radiologists, suggesting that AI systems are ready for responsible implementation in prostate cancer care (Bulten et al., 2022; Saha et al., 2024). However, implementing AI-powered tools for reading MRI pictures from prostate cancer patients does not come without challenges or limitations.

One concern is that technological development challenges, such as biases due to lack of diversity in AI training datasets, will result in overfitting, causing algorithms to fail in predicting future observations based on new data (Thomas et al., 2023). Other concerns include challenges in integrating AI into existing clinical practice (Thomas et al., 2023), and issues related to AI decisions’ transparency and accountability due to its multifaceted nature and sociotechnical structure (Novelli et al., 2024). According to patients in Fransen et al.’s (2025) study, accountability of misdiagnosis set by AI should be shared between hospital, radiologist, and program developers, in descending order. A broader overarching concern is potential negative societal impacts of AI implementation. For example, Alowais et al. (2024) highlight challenges related to data privacy and bias, while Reddy (2024) emphasizes need for conscientious change management and structured, implementation science–based adoption programs for successful, safe, and responsible AI implementation. Hogg et al. (2023) point to another challenge related to implementation of AI-enabled clinical decision support tools (CDSTs) in healthcare, namely, the lack of research on how AI’s potential can be transferred to real-world clinical healthcare practices despite the fact that research communities are aware of and acknowledge the importance of this type of research (Hogg et al., 2023).

Previous research addresses how patients’ earlier experiences with healthcare system and their relationships with healthcare professionals can influence patients’ trust (Mechanic & Meyer, 2000; Ward, 2018), and ultimately their trust in AI tools (LaRosa & Danks, 2018; Lupton, 2018; Richardson et al., 2022). Trust is essential for the healthcare system to function (Rhodes and Strain, 2000), as higher levels of trust increase patients’ likeliness to seek medical care when they need it (Trachtenberg et al., 2005). High levels of trust make patients more prone to accepting treatment they are provided (Hall et al., 2001) and strengthen their ability to disclose personal information in doctor–patient relationships (Gilson, 2003), including details regarding their symptoms (Trachtenberg et al., 2005). Patients’ trust in the healthcare system might ultimately influence their trust in AI tools (LaRosa & Danks, 2018; Lupton, 2018; Richardson et al., 2022), but we do not know what happens or what their potential trust comprises. Thus, the aim of our study is to investigate patients’ trust in AI as part of prostate cancer diagnostics.

## Theory

Previous research on trust in healthcare lacks a clear definition of dimensions and “key attributes” of trust (Taylor et al., 2023). In this study, trust is understood as the general public or patients’ embodied expectations that healthcare systems, institutions, and professionals will act in the best interest of the patient (Davies, 1999; Giddens, 1990; Gilson, 2003). Trust is often compared with acceptance. Acceptance, however, refers to whether patients are likely to consider a medical intervention acceptable and is shaped by elements like institutional reputation, perceived quality of care, and their emotional and cognitive responses (Sekhon et al., 2017). Although some patients might not trust the system, they may still accept the use of AI, due to lack of alternative diagnostic methods or treatment. Another concept related to trust is confidence, which denotes patients’ rational evaluation of the technology’s competence and reliability (Leonardsen et al., 2020). Consequently, confidence in technology can be influenced by how it is communicated and how patients perceive its use in clinical practice.

According to Giddens (1991), trust exists at two different levels: institutional and interpersonal. In this study, institutional trust is perceived as trust in the healthcare system and institution. Interpersonal trust is perceived as trust found on the individual level, in doctor–patient relationships. Previous research shows that the reputation of an organization and its representatives may influence the development of patient trust (Ward, 2018). In this particular context, both healthcare institutions and professionals have a role in establishing patient trust.

Building on this, Giddens (1990) addresses emerging issues related to trust in modern society and describes how modernity dynamically shapes our lives through different concepts, such as contingency and reliability, features that fail or prevail when something or someone faces a situation with an uncertain outcome, leading to uncertainty about what the future might bring. Giddens’ (1990) description of the relationship between trust and risk suggests what can prove to be a challenging exercise for prostate cancer patients; they need to navigate expert systems and new knowledge to balance trust, safety, and risk in their prostate cancer diagnostics.

Giddens’ theory of consequences of modernity offers a valuable lens for understanding disembedding mechanisms and abstract systems that shape modern society and, ultimately, a comprehensive conceptualization of risk and trust in modern society. However, this theory has been subject to critique, as it assumes that modern institutions possess strong capacities for reflexivity and control. Scholars including Gimenez (1992) and Mulinari and Sandell (2009) have problematized Giddens’ tendency to universalize experiences of modernity, overlooking aspects such as gender, race, and class, and how these influence individuals’ encounters with abstract systems. Relevant to our study, for instance, gender, age and class might influence how patients perceive risk and trust.

A key feature essential for modern institutions like the healthcare system to function is the separation of time and space. This separation reconstructs social relations from local contexts to a standardized society and relies on symbolic tokens like money and expert systems, which in turn lays the foundation for development of an abstract level of trust (Giddens, 1990). Expert systems are guarantors for the fulfilment of expectations (Lucas & Gaag, 1991). These systems require a sense of trust based on individuals’ personal knowledge, and the required trust is based partially on “faith” in these systems, such as the healthcare system (Giddens, 1990). In abstract systems, trust is embedded not in the personal relationship between individuals but rather in the reliability of the system itself and its capacity to operate. However, representatives from their respective organizations, experts such as the healthcare professionals, need access points to maintain trust in the institution, through displaying a certain level of trustworthiness and reliability (Fulmer & Gelfand, 2012; Giddens, 1990). Abstract trust could be transferred to how we use technology, as well. Kiran and Verbeek (2010) describe how using technologies assumes that we as humans trust ourselves to the technology and to expert systems.

Kiran and Verbeek (2010) suggest an alternative third perspective: perceiving the relation between human and technology as internal rather than external, arguing that “every technological development puts at stake what it means to be a human being” (p. 1). Trusting ourselves to technology also entails an acceptance of the influence it can have on us (Kiran & Verbeek, 2010). Sauerbrei et al. (2023) bring about yet another interpretation of what implementing AI technologies in the modern healthcare system can entail, suggesting that AI technologies can be perceived as a third actor when used to carry out decision-making processes, with patient and doctor being the other two.

Previous research on AI use in healthcare indicates that patients’ need for extensive validation of AI’s decisions by healthcare professionals (Thomas et al., 2023) is driven by AI-suspicious patients who prefer radiologist involvement in diagnostics (Fransen et al., 2025). Building trust in AI can be facilitated by keeping a “human-in-the-loop” (Holzinger, 2016), in this case a healthcare professional. Holzinger (2016) refers to the advantages of involving humans in the process, for instance, healthcare professionals, to explain AI development and functionality. By serving as humans-in-the-loop, they can demonstrate their trust in AI to reassure AI-skeptical patients, building on trust established in their existing doctor–patient relationships (Starke & Ienca, 2024). Strategies to build this trust include patient-centered communication and shared decision-making (Witkowski et al., 2024), demonstrating reliability and safety, and addressing ethical concerns (Botha et al., 2024).

## Methods

This was a qualitative interview study with men who had been diagnosed with prostate cancer. Qualitative interviews are a well-suited method for research on sensitive subjects. Interviews were exploratory and semi-structured (Gray, 2018). Semi-structured individual interviews allow for establishing connections, creating safe spaces, and making it easier for participants to share vulnerabilities and insights (Pratt, 2021).

### Setting

This study is affiliated with the research project *PROPERMED: Prostate cancer—Personalized medicine powered by MRI and AI*. PROPERMED is an interdisciplinary project with scholars from three faculties at the Norwegian University of Science and Technology (NTNU) and one Norwegian hospital, covering expertise in sociology, health service, AI, MRI, oncology, radiology, and urology. The project aims to explore synergies between technical AI development, prospective clinical testing of AI for prostate cancer detection and biopsy targeting, and evaluation of its societal impact. Partners in the PROPERMED project have developed PROVIZ, an AI-based decision-making support tool, programmed in-house at NTNU, to improve accuracy in prostate cancer diagnostics through analyzing MRI pictures to ultimately improve patients’ quality of life, and to decrease mortality among prostate cancer patients (NTNU Health, n.d.).

This study contributes to the main goal of PROPERMED’s third work package: assessing societal impact of the PROVIZ AI tool.

### Ethical Statement

The project was approved by the Norwegian Agency for Shared Services in Education and Research (SIKT, reference number 798272). All participants signed an informed consent. Interviews were audio-recorded with an encrypted digital recording smartphone application, *Nettskjema Dictaphone* app, and uploaded to an encrypted research server run by the University of Oslo, where they are still saved. Interviews and their corresponding audio files were assigned numbers sequentially to anonymize participants.

### Recruitment and Participants

Prostate cancer patients who had consented to the use of AI in their diagnostic process were invited to the study. Names and addresses of potential study participants were provided by organizers of the PROVIZ clinical proof-of-technology study. Patients received an information letter and a prepaid response envelope. Most of the participants joined the study by returning the consent form by mail, while some signed up by e-mail and phone. Participants were 59–79 years old. All participants had been diagnosed with low-grade or low-risk prostate cancer. Half of participants were working; the other half was retired. Educational backgrounds ranged from secondary school to PhD degree. They were, or had been, employed in both white- and blue-collar jobs.

### Data Collection

Eighteen men participated in the study. All but one interview was done face to face at a meeting room at a university campus, and one interview was conducted by phone. Data collection was carried out during 2024. One participant suffered from hearing impairment and brought two interpreters for assistance. The duration of the interviews ranged from 28 minutes to 1 hour 33 minutes. Audio recordings were transcribed automatically with OpenAI Whisper V2 and V3 and then proofread by the first author.

The interview guide covered four topics: participants’ initial encounter with healthcare institutions and professionals in the prostate cancer diagnostic process; their knowledge and competence about personalized prostate cancer medicine and AI; expectations for treatment and AI; and trust in the Norwegian healthcare system and AI. Topics were developed with prostate cancer patients diagnosed with the assistance of the PROVIZ AI tool in mind.

Prostate cancer is a severe potentially lethal condition, and as prostate cancer patients are considered a vulnerable group, measures were taken to ensure their anonymity. However, reflecting on and considering participants’ situation prior to data collection was equally important to avoid patient discomfort. As qualitative researchers, we must be mindful of the uncertainty and potential negative emotions in the prostate cancer diagnostic process that patients have had to endure. Anyhow, conducting these interviews never felt difficult, perhaps due to participants’ generally positive outlook on their prostate cancer diagnosis and treatment, their free-spoken nature, and their thoughtful reflections on their role in the diagnostic process. Participants also expressed a genuine desire to contribute to research on prostate cancer. All participants actively chose to take part in this study. Notably, none of the participants expressed any signs of discomfort at any point during the interviews—neither spontaneously nor when directly inquired about their comfort level. Even when discussing potentially sensitive topics related to their condition, participants appeared at ease, engaged, and were more than willing to share their thoughts and experiences. This consistent sense of comfort suggests that participants perceived interview setting, topic, and approach as appropriate and safe.

### Data Analysis

Reflexive thematic analysis (TA) developed by Braun and Clarke (2022) was used to analyze the data, through its six recursive phases: familiarization, coding, generating themes, reviewing themes, defining and naming themes, and writing up. The first author familiarized with the content of the interviews by listening to audio recordings and cleaning the AI-generated interview transcriptions. The second and third authors then read transcriptions of two to four selected interviews. The coding process began, followed by individually identifying and generating initial themes and discussions about the initial themes identified by the authors. These discussions revealed similarities and differences in the authors’ preliminary analysis and promoted the establishment of common ground to continue reviewing and renaming themes.

Through close reading and analysis, one overarching topic was developed: trust. Themes were developed and subsequently revised by the first author by going back and forth between the third and fourth steps of reflexive TA: reviewing and generating new themes, respectively. This reflexive approach facilitated the development of three themes allowing for a broad perspective on the overarching topic. The themes are *previous encounters with healthcare professionals as a prerequisite for trust in AI*, *trust in professional expertise*, and *accountable healthcare professionals*. These themes were developed through identifying recurring topics in participants’ statements and creating appropriate codes. The coding process was performed using the NVivo software.

## Findings

We identified three themes from our data: *previous encounters with healthcare professionals as a prerequisite for trust in AI*, *trust in professional expertise*, and *accountable healthcare professionals*.

### Previous Positive Encounters With Healthcare Professionals as a Prerequisite for Trust in AI

Participants had previous experiences with healthcare institutions and individual healthcare professionals that had resulted in positive, or satisfactory, outcomes. These encounters contributed to favorable views on healthcare professionals as a group of experts who use their knowledge and act in best interest of the patient. Thus, healthcare professionals could be trusted to work for patients’ best interests. Benjamin expressed his trust in the following manner:

I was at a [first regional healthcare institution] and underwent surgery for hernia, then I went to [the second regional healthcare institution] to replace a joint in my left knee. And then at [the third regional healthcare institution], it was the shoulder (…) I was very satisfied (…) I think they have very skillful people here at [the fourth regional healthcare institution], so, that’s my impression, yes. (Benjamin)

The quotation reflects a broad range of positive experiences with various healthcare institutions, including the current one, where AI-assisted prostate cancer diagnostics were used. Trust built through these encounters appears to extend to both healthcare professionals and technologies they use, such as MRI. Building on these encounters, participants transfer trust in healthcare professionals and technologies to trust in AI, even though encounters with AI are fewer and farther apart. As participants trust in healthcare professionals and the technologies they use, they trust professionals to use AI as well. Participants suggest that the main prerequisite for acceptance or trust in AI, trust in the healthcare professionals, already exists in the Norwegian healthcare system. Ottar drew attention to this:

Back to the question, the trust in technology, the trust in AI or whatever we use, it’s there. Because it really comes down to trust in the person. (Ottar)

The quotation illustrates how trust in healthcare technologies, including AI, is not built in isolation but is instead an extension of the trust in people who use it. In participants’ previous healthcare encounters, doctors and nurses have built trust through caregiving, a task AI cannot perform. Therefore, AI is generally seen as a tool, an assistant to the doctor, rather than a trustworthy decision-maker. Thus, participants are hesitant to accept AI decisions without involvement of healthcare professionals. Trust in AI is conditional and diminishes without doctor–patient trust, which points to a lack of confidence in independent use of AI and that the human element remained essential. Johan put it into words like this:

Trust in machines is much more fragile, in a sense, than trust in humans (…) [If] artificial intelligence would make the diagnosis (…) I feel that I would consider it, as of today, as a suggestion. (Johan)

Healthcare professionals give patients confidence about safety, which is established through caregiving and doctor–patient encounters. The ability of healthcare professionals to communicate clearly, offer reassurance, and provide thorough explanations was seen as something AI could not replicate. Thomas shared his view on how healthcare professionals can facilitate AI trust or acceptance through trust-inducing interaction with the patient:

Interviewer: Did you feel safe during the process?

Thomas: I felt safe, yes. And it was very much about the interaction with the staff and how they were.

Interviewer: So, they had a reassuring function, or?

Thomas: Yes.

Interviewer: Reassured you in that sense?

Thomas: Yes. Very well explained, very thorough. Very good conversation afterwards, when the results were ready.

The quotation illustrates how the interaction with healthcare professionals contributes to patients feeling safe and reassured, even if the diagnostic process involves the use of new technologies, such as AI. The combination of trustworthy healthcare institutions and professionals further supports participants’ ability to trust in or accept AI. Healthcare professionals can act as mediators to facilitate and amplify trust in AI or AI acceptance by endorsing AI and ensuring safety of AI-powered diagnostics and treatment through oversight and validation. For participants to trust in or accept AI, a technology that they have limited or no knowledge about, healthcare professionals must build on patients’ trust from previous encounters and validate AI’s decisions.

### Trust in Professional Expertise

Throughout the interviews, it was clear that understanding how AI works was non-essential, as trust tended to be related to healthcare professionals’ expertise. Healthcare professionals’ endorsement of AI was often sufficient to trust in it, though there was still a clear preference for professionals to make final decisions in prostate cancer diagnostics. This preference is attributed to healthcare professionals’ ability to explain test results, diagnoses, and treatment options. Participants valued this more than their own technical understanding and knowledge. Ottar based his trust in experts’ ability to explain AI:

I rely on trusting those who are experts. And I have no need to question the assessments they make. So, if they say that they use this [AI] tool, have experience with it, and trust it, my response is: that’s fine. I have no basis to question it, and I must trust this (…) You asked me at the start of the interview why I have trust in general practitioners, urologists and MRI and so on. Well, that’s because it has been explained in a way that aligns with my assumptions, or knowledge, or lack of knowledge. Yes, this seems logical, reasonable. (Ottar)

Technologies like MRI were described as safe and trustworthy health technologies and considered an embedded part of diagnostics in healthcare, unlike AI, which is not yet widely integrated. Trust in MRI and AI increased when healthcare professionals endorsed and explained the technologies in a way that participants understand. Participants expected that healthcare professionals would use their expertise to act as interpreters and explain how AI works and assesses its input, in the same way that MRI has been explained to them. Healthcare professionals should do this to enhance trust in AI. Ottar shared his thoughts on this:

Ottar: But perhaps what we are talking about now demands that healthcare professionals, and they probably have that in their training, it’s important that they are able to explain it to me, as someone who doesn’t have any knowledge about this.

Interviewer: To translate it?

Ottar: To translate it. Develop a language. Translate it. Create a feeling of safety.

Rather than demanding technical detail, participants expected healthcare professionals to use their expertise to explain AI’s functionality, create a shared language to translate AI, and act as intermediaries between participants and AI. Healthcare professionals should develop a common language around AI to explain how it works, build trust, and reassure participants by showing that they understand AI, they know how to use it safely, and they feel confident putting it into practice. When AI was introduced with justification and confidence, it was more likely to be accepted. William reflected on this matter:

Yes, if a trust-inducing person had said it, then … If I had the feeling that I could trust in the person concerned, I probably would have believed more in [AI], yes. But that the person concerned at least justifies it, so that it’s not simply an assertion, or without explanation. (William)

Participants trusted healthcare professionals’ expertise and consequently AI when endorsed by them but still underlined the need to understand how AI works through doctor–patient communication. Receiving information from healthcare professionals, and feeling seen and heard by them, was important for fostering a sense of safety, something participants felt that they would not have had from a diagnosis based solely on AI. At the time of the interviews, autonomous AI in prostate cancer diagnostics was considered unsafe. Consensus was that AI lacked human intuition and holistic perspective that healthcare professionals have, like human ability to see the whole picture through a clinical gaze. In other words, AI was seen as missing the medical expertise that healthcare professionals use to make assessments and the ability to explain AI’s functionality in a way that participants could trust. Johan drew attention to this:

But if there is a parameter that doesn’t exist, and which is very difficult to, for example, quantify of qualitatively assess and so on and so forth, that’s exactly where … It may well be an exaggeration or making the human assessment (…) more important than it really is, but there’s always this [concern]: “is there a residual factor we have failed to consider?” (Johan)

It would be “scary” to accept a diagnosis set by AI autonomously due to potential errors, and participants presume that AI would not be able to explain its assessments, thus making it challenging to foster trust. This is reflected in the statement of Christian:

You probably have to consider that there might be errors as well, that you double check, especially with regards to diagnostics. It can be scary if the computer decides by itself. (Christian)

While there were concerns about AI making mistakes if left to operate independently, the risks of dismissing AI’s recommendations were also problematized, particularly if doing so could result in missed cancer diagnoses. If this were to happen, it could diminish participants’ trust not only in the healthcare professionals and their expertise but also, by extension, trust in the healthcare system as a whole. Alexander articulated it like this:

[If] the doctor said and declared me healthy, [and] artificial intelligence said: oh wait, there’s something we want to investigate further, (I) went back to the doctor [and] the doctor said: no, I’m confident in my assessment, so we’ll leave it at that (…) [The doctor] dismissed the artificial intelligence, a year passed (…) and then the cancer had developed (…) [Then, the] trust in the healthcare system would take a hit. (Alexander)

While healthcare professionals were expected to explain and justify use of AI, there was also an expectation that they would consider AI’s assessments seriously in clinical decision-making, suggesting that healthcare professionals have a dual responsibility. This highlights a deeper dimension of trust: participants expect healthcare professionals to use their own judgment and integrate both their own expertise and AI’s input. Trust in AI in prostate cancer diagnostics is conditional, depending on how healthcare professionals use their expertise to explain and justify it, and whether it is implemented and used responsibly and transparently.

### Accountable Healthcare Professionals

While there was general confidence in use of AI-powered diagnostic tools within the Norwegian healthcare system, there were still concerns about accountability for AI mistakes. Participants preferred a second opinion from healthcare professionals when AI provides an assessment, because holding a human accountable for any wrongdoings, they argued, is easier than blaming a “machine,” the AI-powered technology. This uncertainty made it harder to fully trust in AI, especially in high-stakes contexts like prostate cancer diagnostics. However, by taking responsibility and being accountable for AI’s decisions, healthcare professionals can strengthen trust in both technology and themselves. Participants repeatedly emphasized the need for healthcare professionals to remain the ultimate, accountable decision-makers. Tobias articulated it this way:

You would not have anybody to blame, if the machine made a mistake. But you would have had a human to blame if the doctor made a mistake. (Tobias)

Due to the fear that AI cannot be held accountable in a similar manner as healthcare professionals, it became harder for participants to build trust in AI. Even though there was an expectation that AI would outperform doctors in interpreting MRI images, medical doctors were responsible for the outcome, not AI. The following statement by Oscar represented this view:

But of course, if an AI has assessed one million other cases, and can compare it with the pictures that are taken here, that has to be better than what a doctor can do, as he only has his own knowledge. It’s an assistant tool. That responsibility does not belong to AI; it belongs to the doctor. AI together with the doctor. They might do another assessment, if AI prompts them to. Alright, let’s do it again. For instance. (Oscar)

Healthcare professionals’ and AI’s errors are judged differently. Healthcare professionals were more likely to be forgiven should they make a mistake, as their fallibility was perceived as a natural part of being human. Contrarily, mistakes made by AI were harder to accept, and consequently, trust was harder to rebuild. Jacob shared his opinion on the matter:

Interviewer: Do you think you would have found it easier to trust a human again [than AI]?

Jacob: I think I would have found it easier to … Should we use the term forgive, a human, because it is just, in quotation marks [sic], a human. We have all made a mistake in some context, without it being serious mistakes, but … It could have led to serious mistakes if we had been unlucky (…) We know how thin the line is.

The phrase “to err is human” was a frequently recurring statement, reflecting participants’ tendency to forgive and regain trust in humans quicker than AI. While unsupervised AI could be trusted to handle low-risk assessments such as bone fractures, participants were unwilling to let AI perform prostate cancer diagnostics autonomously because consequences of prostate cancer misdiagnosis were considered more severe. This distinction indicates a direct link between perceived risks and consequences of misdiagnosis and need for accountable healthcare professionals to supervise AI. Ultimately, trust in AI was closely associated with presence of accountable healthcare professionals. Confidence in AI increased with human involvement, human accountability, and use of AI-powered technology as a support tool rather than a replacement for human judgment.

## General Discussion: The Importance of Trust in Healthcare Professionals in AI-Powered Healthcare

Implementation of AI-enabled CDSTs in healthcare is a new aspect of diagnostics and treatment that can that can influence how patients relate to health services as well as various other healthcare services. Investigating this implementation from the patient’s perspective is essential to understand how it affects them. Therefore, we explore how participants express trust in healthcare institutions, professionals and AI.

Our main finding is that trust in healthcare professionals is crucial for acceptance and use of AI tools in prostate cancer diagnostics, as trust in AI relies on trust in healthcare professionals. Participants build trust in healthcare institutions, professionals, and AI through several interconnected processes. We developed three dimensions of trust based on our data. The first dimension is trust built on previous positive encounters, the second dimension is trust built on healthcare professionals’ expertise and ability to explain AI, and the third dimension is trust built on professionals’ willingness to take accountability for AI’s assessments and decisions.

Healthcare professionals are crucial for building AI trust, should the AI assume tasks of healthcare professionals. Trust in healthcare professionals that our participants expressed, we argue, is what Giddens (1991) would denote as trust on an interpersonal level. Our participants have built trust on the interpersonal level through doctor–patient relationships. Participants’ trust in healthcare professionals and AI is composed of different interconnected dimensions of trust. The first dimension is that previous positive encounters of participants build trust in doctor–patient relationships and facilitate trust in new doctor–patient relationships. This finding is supported by Mechanic and Meyer (2000). The participants in their study found that good communicative skills were particularly important for building trust in doctor–patient relationships and that previous positive encounters with doctors taking the time to properly get to know them had a trust-inducing effect (Mechanic & Meyer, 2000). Moreover, the prostate cancer patients interviewed in Lysø et al.’s (2024) study expressed a clear preference for human involvement in prostate cancer diagnostics, as they required empathy in communication that they believed AI would not be able to provide. This further underlines patients’ need for good doctor–patient relationships to build trust.

Trust in doctor–patient relationships is intertwined with the second dimension of trust that we found: trust in healthcare professionals’ expertise. Trust built through previous positive encounters with healthcare professionals creates a foundation for participants’ willingness to accept professionals’ expertise and their endorsement of AI. Healthcare professionals’ expertise extends past technical competence and includes doctor–patient communication, translation, explanation, and justification of AI. Participants deemed healthcare professionals an irreplaceable element of successful AI use and expected them to take on roles as translators and explain AI tools. Participants’ demand for healthcare professionals’ involvement as a prerequisite for AI use, we argue, is what Holzinger (2016) would describe as the expectation that healthcare professionals act as “human-in-the-loop.” Humans in the loop, in this case healthcare professionals, are playing an active role to effectively explain AI, making it clear how it fits into diagnostic processes. This is important because participants demand justification and validation of AI’s assessments to accept its use. Furthermore, healthcare professionals must be part of the loop to use participants’ trust that has been built through doctor–patient relationships and expertise to build specific trust in AI. If healthcare professionals are not present to facilitate trust in AI, participants have no foundation on which to build AI trust. Besides indicating the importance of a good doctor–patient relationship for building patient trust, participants in Mechanic and Meyer’s (2000) study also cited competence as a key factor for building trust, just like participants in our study. Mechanic and Meyer’s (2000) study participants demanded that healthcare professionals use their expertise not only to make assessments but also to explain their decisions to patients, to build trust (Mechanic & Meyer, 2000). These findings align with our study participants’ expectation that healthcare professionals stay in the loop to explain and justify the use of AI.

Participants believe that healthcare professionals will use AI wisely, manage its limitations, and, ultimately, take responsibility for AI’s outcome. Their belief that healthcare professionals will take responsibility for AI’s outcome underpins the third dimension we found: the expectation of accountability. Patients’ trust in healthcare professionals and their expertise creates a foundation for patient confidence that professionals will be accountable, and based on this foundation, our participants will be more open to the use of AI. Participants explicitly stated that healthcare professionals, humans, must make the final decision and be accountable regardless of AI’s accuracy. Just like the majority of our participants, prostate cancer patients in Fransen et al.’s (2025) study expressed a preference for the involvement of healthcare professionals in prostate cancer diagnostics assisted by AI. Despite this preference, more than half of the participants in Fransen et al.’s (2025) study stated that they would accept autonomous AI decisions, should they be proven to outperform radiologists. While the participants in our study expressed a belief that AI already is or will be more accurate than radiologists in the future, they do not accept or trust in autonomous AI in prostate cancer diagnostics, unlike participants in Fransen et al.’s (2025) study. Participants in our study perceived AI-powered tools as a new technology that brings about new types of risk that demands human second opinion. This risk, we argue, is what Giddens (1990) would identify as a new type of “manufactured risk.” Participants expressed a hesitancy to impose what they perceived as unnecessary risk upon themselves by potentially being misdiagnosed by AI. This is because prostate cancer is perceived as a severe condition with high risks related to misdiagnosis. The unwillingness to accept this risk implies that participants do not have sufficient trust that AI requires to be accepted unconditionally. Participants alleviated the risk of AI use by requiring human second opinion, and their acceptance of AI use is based on trust in healthcare professionals.

By showing confidence in the technology, or by trusting in healthcare professionals’ expertise and endorsement of AI, participants found it easier to trust the technology itself. We argue that participants entrust themselves not only to healthcare professionals but also to the technology (Kiran & Verbeek, 2010), albeit with reservations. Participants’ reservations regarding AI and their demand for healthcare professionals’ endorsement of it can be linked to the perception of AI as a new and unfamiliar element in prostate cancer diagnostics. This view, we argue, aligns with what Sauerbrei et al. (2023) would refer to as perceiving AI, as a “third actor.” Entrusting themselves to healthcare professionals and AI, hoping that they will make the right assessment, is a way for participants to cope with risk and insecurity that prostate cancer, a potentially deadly condition, brings about. Moreover, participants consider diagnostics by healthcare professionals and AI in combination safer, hoping it will help mitigate the risk of potential overdiagnosis and any subsequent overtreatment. To be able to accept and trust in the use of AI-powered technologies, participants rely on their existing trust to build specific trust in AI in decision-making processes.

Previous research suggests that healthcare professionals can mediate trust from their respective doctor–patient relationships to AI (Starke & Ienca, 2024), further supporting our finding that healthcare professionals are essential mediators of trust in AI. However, if participants perceive that healthcare professionals are not managing AI properly, for instance, by dismissing AI’s findings resulting in progressing cancer, it damages trust in both the professionals and the broader system’s ability to use technology responsibly. It shows how participants expect professionals to engage with AI appropriately, not just brush it aside. While participants’ high levels of trust in healthcare institutions can influence their trust in healthcare professionals and the AI tools that they use positively, wrongful use of AI tools impacts their trust in healthcare professionals and institutions negatively.

Participants’ trust in AI in prostate cancer diagnostics is fragile and conditional and depends almost entirely on human elements— specifically, trust in healthcare professionals, which healthcare professionals build through positive encounters, expertise-based decisions with satisfactory outcomes, and a willingness to be accountable for their decisions. Healthcare professionals are mediators who must guarantee AI’s assessments and decisions. Development in AI’s accuracy and reliability over the past few years and its acceptance and its trustworthiness in the eyes of the participants in our study dealing with prostate cancer comes back to human relationships. Trust is dependent on healthcare professionals’ ability to mediate trust in AI based on previous positive encounters, using their medical expertise to explain and endorse AI, and taking accountability for AI’s assessments and decisions.

## Strengths, Limitations, and Implications

Our study is one of few doing interviews with cancer patients diagnosed with the assistance of AI, therefore offering valuable insights into patients’ perspectives on AI. Reporting our study, we have been transparent on methodological choices, research context, and conceptual approach, which allow contextual transferability.

A limitation of our study is that several of the participants had limited understanding of what AI is or how it was used in their diagnostic process, which was a barrier for in-depth conversation during their interviews. It could also have influenced how they talked about trust in AI. Generalizing our findings to other cultural contexts, gender groups, or cancer types must be approached with caution, as qualitative generalizations are always case-dependent and should be evaluated in the light of its context (Roald et al., 2021). Another limitation of the study is the composition of participants, who were resourceful Norwegian men, within a limited age range. These contextual aspects may influence how risk and trust are perceived and should be explored further.

Although this study investigates prostate cancer patients at a Norwegian hospital, its relevance extends beyond this specific cancer and national context. Perceptions of risk and trust when AI is introduced in the diagnostic process may also be relevant to patients with other forms of cancer. However, the fact that prostate cancer has high survival rates may have led to participants being more open toward testing new technology in diagnostic processes than patients facing other more lethal cancers.

Effective patient-centered communication is a way to foster doctor–patient trust (Witkowski et al., 2024). Our findings offer insights that can be used to develop communication strategies about AI-assisted diagnostics aimed at patients, for both medical students and healthcare professionals. An implication for practice, based on our findings, could therefore be to implement AI-assisted diagnostics as a theme in patient communication training within medical education. This could enhance AI competence among healthcare professionals and improve patient communication, thereby fostering trust.

### Conclusion

Our study contributes to filling a gap in the field of research on the impact of AI use in prostate cancer diagnostics. It helps us understand how patients diagnosed with the help of AI experience and understand the process, and how they express trust in AI. Our findings underscore the central role of trust, highlighting the complex interplay between abstract trust in institutions, interpersonal trust in healthcare professionals, and the emerging trust, or lack thereof, in AI. Patients rely heavily on the trust they have in their doctors to accept AI, emphasizing the need for healthcare professionals to effectively explain AI, endorse its use, and take accountability for diagnostic outcomes. While patients generally express optimism about AI’s potential, they convey a limited level of trust in AI and are not yet ready to fully trust autonomous AI in critical diagnostic decisions. Inability to trust autonomous AI primarily stems from concerns about accountability and the perception that AI lacks human intuition and a holistic approach. Patients have a greater willingness to forgive human error than AI misdiagnosis. Further research and strategies focused on enhancing AI competence among patients, as well as healthcare professionals, are paramount for ensuring transparency and accountability and for maintaining the human element in AI-assisted healthcare—all of which are essential to enhance patients’ trust in AI-powered technologies.
